# From an Apple to a Pear: Moving Fat around for Reversing Insulin Resistance

**DOI:** 10.3390/ijerph192114251

**Published:** 2022-10-31

**Authors:** Maha Alser, Mohamed A. Elrayess

**Affiliations:** 1 Biomedical Research Center, Qatar University, Doha P.O. Box 2713, Qatar; 2 College of Pharmacy, QU Health, Qatar University, Doha P.O. Box 2713, Qatar

**Keywords:** diabetes, obesity, body fat composition, fat depot, abdominal subcutaneous fat, gluteal-femoral subcutaneous fat

## Abstract

Type 2 diabetes (T2D) is a chronic condition where the body is resistant to insulin, leading to an elevated blood glucose state. Obesity is a main factor leading to T2D. Many clinical studies, however, have described a proportion of obese individuals who express a metabolically healthy profile, whereas some lean individuals could develop metabolic disorders. To study obesity as a risk factor, body fat distribution needs to be considered rather than crude body weight. Different individuals’ bodies favor storing fat in different depots; some tend to accumulate more fat in the visceral depot, while others tend to store it in the femoral depot. This tendency relies on different factors, including genetic background and lifestyle. Consuming some types of medications can cause a shift in this tendency, leading to fat redistribution. Fat distribution plays an important role in the progression of risk of insulin resistance (IR). Apple-shaped individuals with enhanced abdominal obesity have a higher risk of IR compared to BMI-matched pear-shaped individuals, who store their fat in the gluteal-femoral depots. This is related to the different adipose tissue physiology between these two depots. In this review, we will summarize the recent evidence highlighting the underlying protective mechanisms in gluteal-femoral subcutaneous adipose tissues compared to those associated with abdominal adipose tissue, and we will revise the recent evidence showing antidiabetic drugs that impact fat distribution as they manage the T2D condition.

## 1. Introduction

Type 2 diabetes (T2D) is a chronic health condition where the blood glucose level is elevated. T2D is a consequence of insulin resistance (IR), where the body cells are irresponsive to insulin and unable to absorb glucose from the blood. IR is associated with the development of dyslipidemia and inflammation in the affected individuals [1]. Dyslipidemia is defined as a state of imbalance in blood lipids, including elevated levels of triglycerides, smaller and denser low-density lipoprotein than normal, and lower high-density lipoprotein levels [2]. IR also causes high blood pressure and glucose intolerance, which leads, with other factors, to T2D progression [3].

One of the major risk factors for IR and T2D is obesity. Obesity-related T2D has become a global pandemic. It is estimated that affected patients with obesity-associated T2D will reach 300 million by 2025 [4]. This is primarily due to the transition to a more sedentary life in specific countries [5]. Although the relationship between obesity and IR is well established, the causal relationship between obesity and IR, among other metabolic disorders, is more complex. Clinical studies have shown that between 10% and 40% of obese individuals (having a body mass index (BMI) > 30), in fact, are metabolically healthy and exhibit normal insulin sensitivity [6]. On the other hand, both lean and overweight individuals are mostly insulin sensitive, but some can develop metabolic disorders including IR [7]. The explanation for lean individuals developing a metabolically obese state may lie in the body fat content and fat distribution [8].

Body fat content and fat distribution is identified as the total body content of adipose tissue (AT). This specialized tissue is a connective tissue consisting of specialized cells called adipocytes and other types of cells. The health of AT is critical in terms of metabolic disorders, as it plays essential metabolic roles as will be discussed in Section 2 [9]. Dysfunctional AT can cause different pathologies and metabolic diseases, including IR and cardiovascular disorders (CVDs). It is known that AT is not a fixed organ that grows in one specific location, but rather distributed in different locations in the body. These variations are called adipose tissue depots [10]. Different individuals tend to have different fat distribution, as their bodies accumulate fat in different depots more than in other depots. This variation is due to differences in their genetic background or environmental factors, such as diet and exercise [11].

AT is deposited in different depots, mainly abdominal and peripheral. Abdominal fat is classified as either subcutaneous or as visceral fat, while peripheral fat is the subcutaneous fat stored in the body’s peripheries, mainly gluteal-femoral fat. Different depots have different functions, and the ratio between these depots plays a major role in health and disease in a more specific way than total body fat content. Previous research has laid the foundation for different depots and differences in their anatomy, physiology, and pathophysiology, as well as their associations with metabolic disorders [12,13,14]. Abdominal fat accumulation is highly associated with the risk of metabolic disorders, whereas gluteal-femoral fat has been shown to have a protective effect against these disorders. In order to utilize this risk difference in a beneficial way, the fat redistribution concept has been proposed. Fat redistribution could be used with cases having high abdominal fat content. If the accumulated fat is successfully redistributed from the abdominal depot to the gluteal-femoral depot, the risk of developing metabolic disorders could be reversed. In this paper, we review articles that studied these differences between depots, and summarized the evidence suggesting that fat redistribution may be utilized as a therapeutic approach to reverse pathologies like IR.

## 2. Adipose Tissue

AT is a specialized connective tissue; it possesses a high level of heterogeneity in terms of cells. AT consists mainly of fat cells (adipocytes); other types of cells include immature adipocytes (preadipocytes), vascular endothelial cells, macrophages, T-regulatory cells, and mesenchymal stem cells (MSC).

AT expands and develops into obesity in two different modes: hypertrophy (increased adipocyte size) and hyperplasia (increased adipocytes number), or a combination of the two modes. This growth in size and number is mediated by environmental and genetic factors [15]. AT remodeling (expansion and breakdown) occurs in a coordinated way to regulate the dynamics of nutrients and body metabolism at different levels. In the case of excessive nutrient storage, the newly formed AT could become dysfunctional, causing elevated inflammation and IR, which can lead to multiple metabolic disorders such as T2D and CVDs [9].

### Classification and Functions of Adipose Tissue

Previously, AT was classified only into two major types based on function, the white adipose tissue (WAT) and the brown adipose tissue (BAT). Recently, more evidence is suggesting the involvement of other criteria to classify AT, including physiology (function), cell type, secretory, and anatomy/depot, as discussed below.

From the functional point of view, AT is classified into two major subtypes: white adipose tissue (WAT) and brown adipose tissue (BAT). WAT is composed mainly of white adipocytes, and it is the key energy reservoir for other organs [11]. WAT main function is energy storage in the form of triglycerides to be released in the form of free fatty acids under fasting conditions. The brown adipose tissue is composed of brown adipocytes, which are responsible for heat generation and maintaining thermal homeostasis. Additional subtypes are the beige AT, which is composed of brown adipocytes within white AT depot, and the pink AT, which is the subtype that emerges during pregnancy from white adipocytes and functions as milk-producing mammary glands.

AT consists of other cellular species other than adipocytes, and it serves other functions other than fat storage. The heterogeneity is critical for studying metabolic syndromes. An unbalanced AT function can cause metabolic syndrome by secreting specific adipose-derived bioactive molecules and secretions, which have specific metabolic or inflammatory effects on the different organs [16]. It was not until the 1980s that the secretory function of AT was discovered. AT serves as a heterogeneous endocrine organ that cross-talks with other systems and organs, it produces adipose tissue-specific hormones, also known as adipokines, growth factors, chemokines, and cytokines [17]. These secreted adipokines vary between depots, causing a variation in paracrine and autocrine function of specific depots. Secreted adipokines play major roles in different metabolic processes such as oxidation of fatty acids, liponeogenesis and growth of new AT, gluconeogenesis, cellular glucose uptake, insulin signaling, and energy consumption in various tissues [17]. Disturbance in any of these primary functions of AT (fat storage, energy homeostasis, and adipokines secretion) could affect the metabolic balance in the body and lead to metabolic disorders [18].

The physiologically different subtypes and their secretome can differ based on their anatomical locations. Mainly, the distribution of WAT at different anatomical sites is classified into subcutaneous adipose tissue (SCAT) and visceral adipose tissue (VAT). SCAT is when WAT resides underneath the skin, functioning as a heat insulator and protection against mechanical stresses, while VAT is the fat accumulation around the different visceral organs. A large proportion of visceral fat falls in the category of ectopic fat [11]. The latter is the stored portion of triglycerides in tissues other than the specialized AT, including the liver, skeletal muscles, cardiac muscle, and the pancreas. Storage of fat in these ectopic depots is associated with an increased risk of IR [19].

Another anatomical classification divides AT according to location: abdominal and gluteal-femoral depots. The abdominal fat depot includes both SCAT and VAT. The other main fat depot is the gluteal-femoral fat depot, which is majorly composed of subcutaneous fat with a minority of intramuscular fat composition [8]. Unlike the abdominal fat, the gluteal-femoral fat is not associated with an increased risk of IR [20]. This classification is critical from a clinical point of view, as emerging research is showing that body fat distribution could serve as a better indicator of metabolic disease risk as compared to total body weight [21]. The BMI includes total body weight and is being used as a parameter in clinics, but it has limitations in accurately representing the complexity of obesity and other health complications [21]. A more precise way to assess excess fat storage would be to measure fat content in different depots [22]. The association between these depots and metabolic disorders will be discussed in detail in the next section.

## 3. Adipose Tissue Depots and Risk of Insulin Resistance and Cardiovascular Disease

AT distribution and fat accumulation into different depots is associated differently with metabolic disorders due to their different physiological profiles [23]. Fat distribution and body shape are clinically important and represent a more powerful tool for assessing the risk of metabolic disease than measuring total body weight.

In the early 80s, a Swedish team generated a simple index to assess fat distribution (waist to hip ratio). In this index, the crude measurement of the waist and hip are taken into consideration to generate the final measurement value. The higher the ratio (reflecting high abdominal proportion compared to gluteal-femoral), the higher the risk of developing metabolic disorders including IR, T2D, and CVDs [24,25]. The same conclusions were also presented by another group, which showed a tight correlation between body shape and metabolic complications [26]. These early publications laid the foundations of this concept.

In the late 80s, a research group showed associations between more body measurements and metabolic disorders, mainly IR development. Freedman & Rimm, 1989, analyzed a cohort of females having a matching BMI but different body measurements, and associated them with their IR status. Their main conclusion was that diabetes was inversely correlated with lower body (thighs and ankles) fat accumulation as compared to upper body (waist, bust, and neck) fat accumulation. This is important, as it shows that lower body fat (thighs/ankles) behaves differently than upper body fat (waist/bust/neck) [27]. However, until now, these papers did not involve a cellular/physiological explanation for these findings.

In 2004, Okura et al. reported further evidence on this topic. They showed that increased AT accumulation in the leg could have a cardioprotective effect for the individual [12]. Goss & Gower conducted a study in 2012 which provided evidence that intra-abdominal AT is associated with glucose intolerance and its complications (IR and T2D), while thigh AT is positively linked to a better glucose and lipid metabolism. However, they could not prove whether body fat distribution has a causal association with this metabolic disorder or if there is an upstream process that affects both fat distribution and IR progression [28]. Other papers confirmed these findings. According to Meisinger et al., 2006, overall abdominal obesity is highly correlated with the risk of T2D in both males and females [13]. Additionally, Mclaughlin et al., 2011, showed that a group with IR was found to have significantly higher abdominal fat than their BMI-matching insulin-sensitive (IS) individuals, whereas thigh fat was shown to be significantly lower compared to the IS groups [14]. Although it was shown that body fat distribution is a major player in predicting metabolic disorders risk, this effect is not the same among different ethnic groups. The correlation of IR progression with abdominal fat distribution was compared among different ethnic groups in multiple studies. One study showed an increased risk of IR with abdominal obesity in Pakistani individuals, whereas the association was less evident in Norwegian individuals [29]. This may suggest that a genetic component could influence the association between body fat distribution and IR differently in different ethnicities.

The association of obesity with other metabolic syndromes such as CVD is also well established. More evidence is showing that visceral abdominal obesity, rather than general obesity, is what causes this increased risk [30]. However, fat storage in the lower body depot (thigh fat) can provide a protective role from lipotoxicity caused by high visceral fat [10]. Many studies have focused on the differences between abdominal subdepots and their association with CVDs. The high accumulation of VAT, but not SCAT, is highly associated with CVD risk in elderlies [31]. This difference is most likely due to the difference in physiology between these abdominal depots. VAT, compared to SCAT, contains more inflammatory and immune cells, lower adipogenesis capacity, and higher proportion of larger adipocytes. Adipocytes in VAT are more active, they have a greater capacity to generate and secrete fatty acids, uptake glucose, and generally they are more insulin-resistant as compared to SCAT [32]. Few studies have reported evidence that gluteal-femoral fat exhibits a protective role against CVDs and T2D [20]. Gluteal-femoral depot sequester lipids to be stored in that depot instead of being directed to ectopic fat depot, leading to protection against metabolic disease. These effects were nicely summarized in details in a review by Karpe and Pinnick [33]. In general, the inverse relationship between abdominal and thigh fat with risk of metabolic syndrome, including IR and CVD, may suggest a therapeutic approach involving induction of fat redistribution in high-risk individuals from the abdomen to the thigh, which is discussed in more detail in the following section.

## 4. Fat Redistribution: A Therapeutic Strategy to Reverse Insulin Resistance

Fat redistribution is the process where fat storage is shifted from one depot to another. In the context of reversing IR and its metabolic complications (T2D and CVDs), fat redistribution from the abdomen to the gluteal-femoral depot is getting researchers’ attention as a potential therapeutic strategy. Some existing compounds/IR medications have been shown to affect the formation of new fat and the process of fat distribution as one of their modes of action, leading to fat gain/loss in diabetes patients. In this section, these compounds and their effect on fat redistribution as a treatment of IR will be reviewed and discussed.

### 4.1. Thiazolidinedione (TZD)

One important compound used to treat IR and T2D is Thiazolidinedione (TZD), also known as pioglitazone. This medication is used to reverse the IR state and lower blood glucose levels in T2D patients. Previously, it was believed that a major side effect of using TZD for T2D patients is body weight gain. However, some clinical evidence shows that the fat is redistributed in a beneficial direction from visceral to subcutaneous fat depot [34]. Other clinical evidence showed that the increase in body weight after TZD treatment was associated with a neutral/decrease in visceral fat and abdominal obesity [35]. It is important to mention that the use of TZD requires to be monitored closely, especially when prescribed for patients with high risk of cardiac diseases. This is because it can be associated with side effects, including weight gain due to fluid retention rather than fat distribution, which may progress to cause cardiac complications [36].

The mode of action of TZD is mainly through stimulation of the expression of the master regulator of adipogenesis: Peroxisome proliferator-activated receptor gamma (PPARg) [37]. The exact mechanism of TZD for reversing IR is still not fully understood. However, we know it induces PPARg-mediated expansion of subcutaneous AT, which leads to a drop in systemic fat content and reverses lipotoxicity resulting from fat storage in ectopic depots [38]. Recent clinical studies have shown that TZD treatment leading to AT expansion may be due to the formation and growth of new adipocytes (hyperplasia) in subcutaneous AT depots [39]. This is consistent with the in vivo findings of de Souza et al., 2001, showing that TZD treatment leads to an increase in the proportion of small adipocyte population in subcutaneous AT (hyperplasia) in lab rats [40]. Another important effect of TZD is its anti-inflammatory action, as it activates anti-inflammatory pathways in obesity-associated AT inflammation [41]. One recent investigation showed a wide characterization of the chronic effect of TZD treatment on human AT. The study showed that the glycerophospholipid pool is a major player in how the AT responds to TZD treatment, emphasizing a potential role of adipose cells membrane remodeling as a target of T2D treatment [9]. The findings about TZD mode of action and effect on fat storage are summarized in Table 1. According to previous studies, redistribution of fat storage from the ectopic and visceral anatomical locations into the newly formed subcutaneous depots exhibits a protective effect against IR and its metabolic complications. However, more studies are needed to show if TZD can be used to redistribute fat into the gluteal-femoral fat depot and the therapeutic effectiveness on reversing IR.

### 4.2. Metformin

Biguanide metformin is one of the most widely used medications for blood–glucose lowering and IS improvement in T2D patients. With its good safety profile, it has become the first-line medication for T2D [61]. Studies have shown a clear effect of metformin on reducing body weight and preventing obesity in T2D patients [62], making it the first candidate drug to be used for obese diabetic patients [63]. The weight-lowering effect of metformin was reported to differ among patients with different BMIs. In lean diabetic patients, metformin does not cause a significant weight loss, whereas in obese patients it does; yet, the underlying mechanism behind this difference is still unclear. Studies have suggested that metformin affects body weight through directly affecting adipogenesis. According to Alexender et al., metformin inhibits adipogenesis in vitro using the murine adipose cell line 3T3L1 [43]. However, limited research was done to assess how metformin regulates the adipogenesis-signaling pathways. Another study showed that metformin, combined with insulin treatment, led to enhanced adipogenesis in vitro [43]. Recent investigations conducted on the 3T3L1 murine cell line showed that metformin at low doses (1.25 and 2.5 mM) significantly induced adipogenesis and fat accumulation in these cells, while higher concentrations (5 and 10 mM) had the opposite effect, as they significantly reduced adipogenesis levels in the cells [61].

Studies involving animal subjects and human subjects suggested that metformin specifically affects visceral fat mass. According to Tokubuchi et al., 2017, metformin reduces visceral fat accumulation compared to controls. This effect may not be caused by fat redistribution from visceral to the gluteal-femoral depot, but rather because it elevates the fat metabolism in that depot [44]. Metformin upregulates fat-oxidation-related enzymes, the uncoupling proteins 1 and 3 (UCP1 and UCP3) indicating increased fat burning as a source of energy, causing visceral total mass reduction. This beneficial effect may be the reason why previous studies reported an effective fat loss after using metformin [44]. These findings are summarized in Table 1. Although it has not been demonstrated, the effect of metformin on the size of gluteal-femoral may be worth investigating as an additional added benefit of metformin on reversing IR.

### 4.3. Glucagon-like Peptide-1 Receptor Agonist (GLP-1RA)

The glucagon-like peptide-1 receptor agonist (GLP-1RA) is a class of medications used to control T2D. It has led to a significant improvement in glycemic parameters of diabetes patients. The medication functions by activating the GLP-1 receptors in the pancreas, leading to enhanced insulin release, decreased glucagon release, and enhanced glucose homeostasis [45].

This medication shows other effects on obese diabetic patients, as it leads to general weight loss, which is often dealt with as a positive side effect of GLP-1 and similar medications [64]. Different investigations suggested that GLP-1 has an effective effect by inhibiting adipogenesis (antiadipogenic effect) and enhancing lipolysis (prolipolytic effect) [46].

The effect of GLP-1 on fat redistribution was unclear until very recently. According to Morano et al., 2015, GLP-1 reduces the total fat content and BMI of patients. More importantly, the study showed a difference in fat reduction between different depots [50]. GLP-1-treated cohort showed a significant reduction in both subcutaneous and visceral abdominal fat depots, suggesting a selective antiadipogenic and prolipolytic effects of the drug [50]. This effect is occurring as a result of GLP-1 on adipogenesis and adipogenic pathway markers. According to Challa et al., 2012, in vitro GLP-1 treatment on 3T3L1 cells downregulates PPARg, C/EBPb/d, and regulates the AKT pathway [47]. In human cell lines, GLP-1 affects adipocyte differentiation levels and decreases adipogenesis by downregulating lipogenesis genes and upregulating critical genes in lipolysis pathways [49]. Similarly to metformin, GLP-1 was also shown to decrease body weight by enhancing energy metabolism using fat as fuel [49]; however, its effect on the size of gluteal-femoral also remains to be studied. The findings about GLP-1 are summarized in Table 1.

### 4.4. Sodium-Glucose Cotransporter-2 Inhibitors (SGLT-2 Inhibitors)

Multiple medications fall under the sodium-glucose cotransporter-2 inhibitors (SGLT-2 inhibitors), such as dapagliflozin and canagliflozin. Dapagliflozin is a T2D medication used either alone or in combination with other drugs such as metformin. It is a selective SGLT-2 inhibitor, a major gut and renal sodium-glucose cotransporter. The mode of action of its glucose-lowering effect is by targeting SGLT2, reducing glucose reabsorption in the kidneys. This allows the high glucose content in the blood to be exerted from the body [51]. Many reports showed the effectiveness of dapagliflozin as an antidiabetic drug, but few recent studies showed the link between its insulin sensitization effect and body fat distribution. According to Merovci et al., 2014, dapagliflozin treatment for two weeks improved the condition of T2D patients and decreased their muscle IR, as compared to placebo control [65]. Another study involved the animal model (rats) that showed a positive effect of dapagliflozin on normal and diabetic rats [66].

A recent study showed that treatment of dapagliflozin for four months led to a significant reduction in total body weight and general fat content of their cohort, with no indication of fat distribution [52]. A more recent study reported the effect of dapagliflozin on blood glucose levels as well as body shape and body fat distribution. According to Sun et al., 2020, the treatment of their study cohort with dapagliflozin in combination with metformin for 12 months was associated with enhanced control of their blood glucose levels, indicating an enhanced IS state. Additionally, the study reported that this treatment slightly reduced waist/hip ratio, which indicates a lower abdominal depot fat content as compared to gluteal-femoral depot [53]. Although the mode of action of dapagliflozin as an inhibitor of SGLT2 inhibitor is not yet clear in terms of body fat distribution, the findings indicate that it might affect fat content by influencing adipogenesis. More studies need to be conducted to clarify how it affects adipogenesis at the molecular level.

Canagliflozin is another member of the SGLT2 inhibitors family of T2D drugs that relies on decreasing glucose reabsorption in the kidney [67]. The effect of canagliflozin therapy on T2D and fat distribution was assessed in a few recent studies. One study showed the effect of canagliflozin for 12 weeks on T2D patients. It showed no effect on waist/hip ratio. However, when comparing the subcutaneous and visceral abdominal depots, the study found a significant change in visceral adipose content as compared to baseline level, as well as to the control group. In addition, other parameters like the Homeostatic Model Assessment of Insulin Resistance (HOMA-IR) and Nitric oxide (NO) were improved after the treatment, which indicates an improved IR state and endothelial function [54]. These effects are similar to the effect of dapagliflozin, as both drugs fall into the same family, although their mode of action related to improving fat content and decreasing visceral fat depot remains unclear. The findings about both SGLT-2 inhibitors are summarized in Table 1.

### 4.5. Insulin

T2D is characterized by both IR, and in later stages, defected insulin secretion due to beta cell dysfunction [58]. Oral medications are clinically used to manage the IR condition to control the elevated glycemic level. For many patients, however, T2D proceeds to the stage where insulin secretion is not sufficient. Therefore, insulin itself is used as a therapeutic molecule in combination with other oral medications to treat T2D [55]. A rare complication of the use of insulin-injection treatment is lipodystrophy, where the repeated injection of insulin at the same site causes the subcutaneous fat to be damaged and take a retracted scar shape [68].

The association between insulin therapy and increased total body weight is well established [60]. In 1998, a preliminary study showed that insulin treatment in T2D patients leads to increased total body weight due to increased subcutaneous, but not visceral, fat deposition. Gluteal-femoral fat was not assessed in the study [69]. Animal studies conducted on rats confirmed the same effect of insulin treatment on abdominal fat distribution via increasing subcutaneous fat with no change in visceral fat content [57].

Insulin treatment influences adipogenesis-related processes. A study showed that insulin treatment of human-derived adipose stem cells leads to their enhanced proliferation [58]. It also upregulates differentiation into adipocytes via Wnt signaling pathway inhibition and the downstreatm regulation of adipogenesis markers, including PPAR-γ and CEBP-α [58]. These effects do not conclusively prove/disprove the effect of insulin on fat redistribution. Limited research has been conducted on the effect of insulin treatment on adipogenesis, fat distribution, and body shape. No previous studies have shown the effect of insulin treatment in a depot-specific manner, and this area still needs to be explored due to its clinical relevance for T2D patients on insulin injections. The findings about insulin are summarized in Table 1.

### 4.6. Sulfonylureas

Sulfonylureas are a group of oral medications for T2D, often prescribed as a second line of treatment after metformin medication fails to control the elevated glycemic blood levels [70]. It works by increasing the levels of insulin release from the pancreas if the T2D patient is at a stage where insulin is not sufficiently secreted [70].

Sulfonylureas acts by stimulating the beta-cells in the pancreas to enhance the endogenous levels of insulin and manage T2D [59]. Thus, this treatment is associated with weight gain in a similar way to insulin injection. This limits the use of both sulfonylureas and insulin with overweight and obese patients as summarized in Table 1 [60].

No previous research directly connected sulfonylurea treatment with adipogenesis process, body shape, or body fat distribution. However, based on what we know, sulfonylureas increase insulin secretion, which has been shown to enhance adipocyte proliferation and upregulates adipogenesis differentiation [58].

## 5. Conclusions

Body fat distribution was shown by multiple studies to have a tight association with metabolic disorders, including IR and T2D. Different medications that are currently used to treat T2D were shown to influence fat distribution as one of their modes of action or associated side effects. TZD, metformin, and GLP-1RA impact adipogenesis by targeting adipogenic markers, leading to reduced visceral fat mass and increased metabolism. On the other hand, SGLTs (Dapagliflozin and Canagliflozin) exhibit a positive impact on visceral fat reduction and fat redistribution, their effect on adipogenesis is not yet established. Other T2D critical medications (insulin and sulfonylureas) are associated with significant body weight gain through increased subcutaneous but not visceral fat deposition, but not enough evidence exists on their impact on fat distribution. The beneficial effect of these drugs on improving IS and reversing IR may be partially explained by inducing fat movement from an apple-shaped abdominal obesity to a pear-shaped peripheral obesity. Studies of other drugs that target adipogenesis and fat distribution as an antidiabetic therapeutic solution are warranted.

## Figures and Tables

**Table 1:** Summary of Type 2 diabetes medications, their mechanisms of action, and their effect on total body weight and fat distribution.

T2D Medication	Mechanism of Action	Effect on Total Body Weight	Effect on Fat Redistribution	Study Data Type
TZD	Adipogenesis upregulation through PPARg [37].	Increase [42].	Increased fat in gluteal-femoral depot [34]. Neutral/decrease in abdominal depot fat [35]	Clinical Clinical
Metformin	Adipogenesis downregulation [43]. Fat metabolism enhancement through UCP1 and UCP3) [44].	Decrease in obese T2D patients [42].	Decrease in visceral abdominal depot fat	Clinical
GLP-1RA	Pancreatic GLP-1 receptors activation to enhance insulin secretion [45]. Adipogenesis downregulation through PPARg, C/EBPb/d and AKT [46,47]. Lipolysis upregulation [46,48]. Fat energy metabolism enhancement [49].	Decrease [50].	Reduction in both subcutaneous and visceral abdominal fat depots [50].	Clinical
SGLT2 inhibitors	SGLT2 inhibition to reduce glucose reabsorption in the kidneys [51].	Decrease [52].	Slight reduction in waist/hip ratio (decreased abdominal size compared to gluteal-femoral size) [53]. Reduction in visceral fat content [54].	Clinical
Insulin	Compensates for the reduced insulin secretion [55].	Increase [56].	Subcutaneous, but not visceral fat deposition [57,58].	Animal (rat)
Sulfonylureas	Insulin secretion enhancement [59].	Increase [60].	Not reported.	N/A

T2D: type 2 diabetes; PPARg: Peroxisome proliferator-activated receptor gamma; TZD: Thiazolidinedione; UCP: uncoupling protein; SGLT2: Sodium-glucose cotransporter-2; GLP-1RA: Glucagon-like peptide-1 receptor agonist.
